# A systematic review and critical analysis of cost-effectiveness studies for coronary artery disease treatment

**DOI:** 10.12688/f1000research.13616.2

**Published:** 2018-07-03

**Authors:** Victoria McCreanor, Nicholas Graves, Adrian G Barnett, Will Parsonage, Gregory Merlo

**Affiliations:** 1 Institute of Health & Biomedical Innovation, Queensland University of Technology, Brisbane, Queensland, 4059, Australia; 2 Capital Markets Cooperative Research Centre, Sydney, New South Wales, 2000, Australia; 3 Royal Brisbane and Women’s Hospital, Brisbane, Queensland, 4029, Australia

**Keywords:** Coronary artery disease, cost-effectiveness analysis, economic analysis, review, health policy, health services research

## Abstract

**Background**: Cardiovascular disease remains the primary cause of death among Australians, despite dramatic improvements in overall cardiovascular health since the 1980s. Treating cardiovascular disease continues to place a significant economic strain on the Australian health care system, with direct healthcare costs exceeding those of any other disease. Coronary artery disease accounts for nearly one third of these costs and spending continues to rise.

A range of treatments is available for coronary artery disease yet evidence of cost-effectiveness is missing, particularly for the Australian context. Cost-effectiveness evidence can signal waste and inefficiency and so is essential for an efficient allocation of healthcare resources.

**Methods:** We used systematic review methods to search the literature across several electronic databases for economic evaluations of treatments for stable coronary artery disease.  We critically appraised the literature found in searches, both against the CHEERS statement for quality reporting of economic evaluations and in terms of its usefulness for policy and decision-makers.

**Results:** We retrieved a total of 308 references, 229 once duplicates were removed. Of these, 26 were excluded as they were not full papers (letters, editorials etc.), 55 were review papers, 50 were not cost-effectiveness analyses and 93 related to a highly specific patient sub-group or did not consider all treatment options.  This left five papers to be reviewed in full.

**Conclusions:** The current cost-effectiveness evidence does not support the increased use of PCI that has been seen in Australia and internationally. Due to problems with accessibility, clarity and relevance to policy and decision-makers, some otherwise very scientifically rigorous analyses have failed to generate any policy changes.

## Introduction

Cardiovascular disease is the primary cause of death for Australians and places enormous strain on the health care system. Treatments for cardiovascular disease consume 12% of Australian health care spending, AUD $7 billion annually, with coronary artery disease responsible for 27% of the cost ^1^. Australian health services continue to increase spending in this area, with cardiovascular disease treatment costs doubling between 2000–01 and 2008–09 ^1^. This increase in spending is occurring despite improvements in the cardiovascular health of Australians, resulting from improved lifestyle factors, most importantly reduced rates of tobacco smoking ^2^.

There have been changes in the preferences for different treatments. Since 1998, percutaneous coronary intervention (PCI) has overtaken coronary artery bypass graft (CABG) as the most common revascularisation procedure in Australia ^3^. Between 2000–01 and 2007–08 the number of PCIs performed increased by 57% ^4^. Much of the increase is relates to patients treated for acute myocardial infarction, however there was a 21% increase in PCI performed in patients without acute myocardial infarction ^5^. In 2012–13, 93% of PCIs involved the insertion of one or more stents ^4^. Accompanying the increase in PCIs, there was a 19% reduction in the number of CABGs performed ^6^. Since then, rates of PCI have remained high ^4^. The increase in PCI also suggests that more patients who in the past would have been treated conservatively, with medical therapy only, are now also undergoing PCI.

Invasive treatments for coronary artery disease are costly, involving surgery, expensive equipment and consumables, yet there is no adequate assessment of the cost-effectiveness of the treatments provided. An important question is whether the extra costs incurred are adequately compensated by gains to health. In an era of non-increasing health budgets, changes to practice should be accompanied by improvements in health outcomes, particularly if increased costs are involved. Cost-effectiveness evidence should be used to assess whether changes in costs are justified by changes to health outcomes associated with new services or changes in practice.

In the case of treatment for stable coronary artery disease, it is not clear if there is sufficient evidence to support the recent changes in treatment preferences from CABG to PCI, or a move from medical therapy alone to more invasive treatment such as PCI. While many economic evaluations have been undertaken, much of the literature assessing the cost-effectiveness of coronary artery disease treatments compares only two options at a time, with a large focus on the differences between drug-eluting stents (DES) and bare metal stents (BMS). However, comparing only two treatments at a time is limited. It assumes the chosen baseline comparator is a good quality service, and omits other available treatment options. It is sub-optimal for high-level budgetary decisions to be made without more comprehensive information about all competing treatment choices. An analysis comparing BMS only with DES fails to consider treatments other than stents and may therefore overestimate the cost-effectiveness of one type of stent, over other treatments.

Clinical trial evidence has failed to show a mortality benefit of PCI over medical therapy in the treatment of stable disease, but there is some evidence of greater symptom relief ^7– 10^. Coronary artery bypass graft surgery on the other hand has been shown to provide mortality benefit in some circumstances, and more prolonged symptom relief compared with PCI ^9, 11^. However, PCI is an expensive procedure, and CABG even more so. The question, therefore, is whether the additional costs are sufficiently offset by the greater symptom relief afforded by PCI and mortality benefit and symptom relief of CABG, when compared with medical therapy alone.

The aims of this review are to evaluate the literature that describes the cost-effectiveness of all treatment options for stable coronary artery disease: PCI including stent insertion, CABG and medical therapy, and then to critique the literature based on the quality of the cost-effectiveness evaluations and usefulness of the findings of the research for real-world applications. Usefulness for real-world applications includes the applicability of the outcomes for informing decisions about the allocation of resources, particularly in the Australian context, and the ability to translate the findings into practice.

Prior to undertaking any new research, it is important to undertake a review of the literature, to reduce the chance of duplication of effort, and to avoid tackling questions that have already been answered ^12^. This review is designed to identify gaps in the knowledge about the cost-effectiveness of treatments for coronary artery disease, and therefore to inform future research in this area. Our goal is to provide insights useful for clinicians, healthcare service budget holders, and policy-makers about the best use of scarce resources for the treatment of coronary artery disease.

## Methods

The literature published between January 1995 (after the use of stents was approved in the United States in 1994) and May 2017 was searched in PubMed, Embase, Scopus, CINAHL (via Ebscohost) and EconLit (via ProQuest). The searches focussed on extracting papers that examined the cost-effectiveness of PCI (including stent insertion), CABG and medical therapy together. Due to the large volume of research on coronary artery disease, searches were limited to subject headings where possible. A slightly broader approach was taken to capture medical therapy as this is described less consistently in the literature. Only research published in English was included. The search terms used are in Box 1. There is no specific MeSH for cost-effectiveness analysis and the suggested heading is *Cost-benefit Analysis*. Search terms were modified slightly to fit the subject heading structure of each database (See Supplementary File 1).

Box 1. **Search terms used for PubMed**

(percutaneous coronary intervention[MeSH Terms] OR stents[MeSH Terms]) AND coronary artery bypass[MeSH Terms] AND (Cost-benefit Analysis[MeSH Terms] OR Models, economic[MeSH Terms]) AND (((medical OR conservative) AND (therapy OR treatment)) OR primary prevention OR secondary prevention)

Search results were imported into EndNote X7 software, duplicates were removed and articles then reviewed according to the inclusion and exclusion criteria outlined in Table 1.

**Table 1.** Inclusion and exclusion criteria for review.

Inclusion criteria	Exclusion criteria
**• Full publication or manuscript available** **• Assessed percutaneous intervention, coronary artery** ** bypass graft surgery and medical/conservative therapy** ** together** **• Conducted a full economic evaluation which valued** ** both costs and benefits of different treatments** **• Meta-analyses of cost-effectiveness studies** **•Written in English**	• Cost-analysis only • Compared only two interventions (e.g. stents v bypass graft surgery) • Limited to a highly-specific group of patients (e.g. HIV or critically ill patients) • Editorials, letters, opinion pieces • Reviews • Not actual cost-effectiveness analysis (e.g. methods or protocol papers) • Evaluated screening, diagnostic or rehabilitation, rather than treatment • Conference paper abstracts where full analysis not available

We used the Preferred Reporting Items for Systematic Reviews and Meta-Analyses (PRISMA) Statement ^13^ and checklist in retrieving and reviewing articles for inclusion in this review. Due to the nature of this review, not all items were relevant. Our completed checklist is available in Supplementary File 2. Titles of all papers were reviewed and the abstract or full text examined in detail where required to assess against inclusion and exclusion criteria.

We used the Consolidated Health Economic Evaluation Reporting Standards (CHEERS) Statement ^14^ to assess the quality of reporting of the economic analyses ( Supplementary File 3). The CHEERS Statement and checklist was developed to improve and promote quality of reporting health economic evaluations ^15^. Economic evaluations are designed to assist in health service decision-making and resource allocation. Therefore, due to the opportunity costs of acting on poor-quality evidence, it is particularly important to ensure high-quality reporting in economic evaluations ^15^. The CHEERS Statement checklist consists of 24 items which should be reported and guidance regarding the specific details required. We extracted information from each of the included studies for each item on the checklist, to assess the quality of reporting.

In addition to high-quality reporting, economic evaluations need to be useful to decision-makers, as their purpose is to provide evidence to improve the efficiency of use of healthcare resources. Decision-makers need to understand the potential impact of acting on cost-effectiveness evidence and making changes to healthcare services; most notably what will be the effect on health outcomes and costs, and how certain are these projections? To assess the usefulness of the evaluations for decision-makers, we also extracted data on the interventions compared, the effectiveness measures, whether the analysis applied to a specific patient group, the structure or type of analysis used and the time period of the analysis. We then assessed usefulness of the reporting by rating the reporting of outcomes, costs, uncertainty as: useful, not useful, or partly useful, based on whether the results could be used by a decision-maker. We also looked for a clear statement about the policy implications or direction that should follow based on the outcomes, and gave each paper an overall usefulness rating of low, medium or high, depending on the other elements assessed. We acknowledge that these ratings are subjective and have not been validated, nevertheless we think they are practically useful.

## Results

**Figure 1.**
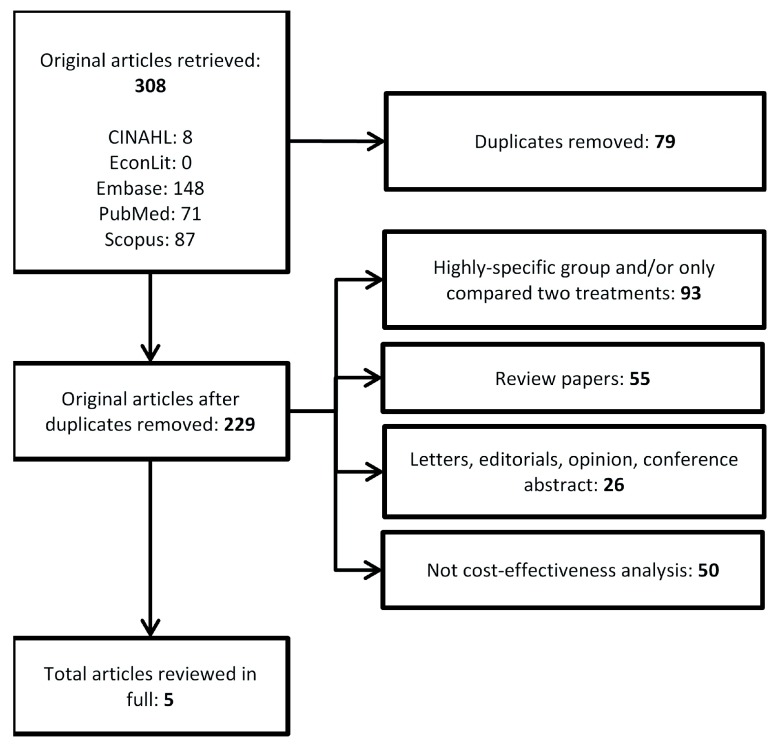
PRISMA Flow diagram of literature review process.

Searches in all databases, except EconLit, revealed potentially relevant articles. A total of 308 results were retrieved, and 229 remained after duplicates were removed. The numbers of papers retrieved from each database are in Figure 1.

Many articles tagged under the cost-benefit or cost-effectiveness subject headings were not cost-effectiveness analyses but simply mentioned cost-effectiveness as a factor for consideration. In addition, most of the cost-effectiveness papers did not examine medical therapy, percutaneous coronary intervention and bypass graft surgery together, but focussed on only two treatments. Papers were excluded for other reasons including a focus on rehabilitation following cardiac procedures, screening of cardiac patients, being commentary only or reviews. However, we checked for papers included in reviews that had not been found in our searches. The results of the review process are shown in Figure 1.

The results of the review process left only five papers for consideration. Table 2 provides a summary of the papers included in the full review. The results varied across the five studies, but across the scenarios analysed most concluded that medical therapy was the cost-effective treatment, and three concluded that CABG was cost-effective. In no scenario was PCI reported as being cost-effective compared with the alternatives ( Table 2). Three studies included quality of life measures in at least part of their analyses and only two studies undertook projections over the lifetime of patients ( Table 2).

**Table 2.** Summary of cost-effectiveness results reported. Most papers reported more than one timeframe. Shading indicates treatment reported as cost-effective.

Author	Year	Effectiveness measure	Timeframe of analysis	Cost-effective treatment		
				OMT	PCI	CABG
**Caruba *et al.*^16^**	2014	Death, MI	1 year, costs only			
			3 years, costs only			
Fidan *et al.*^17^**^^	2007	LY	10 years			
**Griffin *et al.*^18^**	2007	QALY	6 years, clinically appropriate for PCI			
			6 years, clinically appropriate for CABG			
			6 years, clinically appropriate for both PCI & CABG			
**Hlatky *et al.*^19^**	2009	LY, QALY (sensitivity analysis)	4 years			
			Lifetime			
**Vieira *et al.*^20^**	2012	QALY, event-free survival	5 years, event-free costs			
			5 years, event-free plus angina-free costs			

* CABG = coronary artery bypass grafting, LY = life years, MI = myocardial infarction, OMT = optimal medical therapy, PCI = percutaneous coronary intervention, QALY = quality-adjusted life-year. * *N.B. Fidan et al. ^17^ *paper did not make direct comparisons of the relevant treatments*

### Quality of reporting

A summary of the assessment against the CHEERS Statement are shown in Table 4. Overall, the quality of reporting was high, with studies adequately reporting against 50 to 100% of relevant items on the checklist ( Table 3). A more detailed table is available in Supplementary File 3.

**Table 3.** Results of analysis against CHEERS Statement.

Author	CHEERS items satisfied	Relevant CHEERS items	Percent satisfied
**Caruba *et al.*^16^**	23	23	100%
**Fidan *et al.*^17^**	22	23	96%
**Griffin *et al.*^18^**	23	24	96%
**Hlatky *et al.*^19^**	21	24	88%
**Vieira *et al.*^20^**	12	24	50%

Due to their nature, not all CHEERS items were relevant to all studies.

**Table 4.** Summary data extracted for assessment of usefulness of included papers.

Author	Year	Country	Disease severity/ Patient group	Comparators	Effectiveness measure	Model	Time period
**Caruba *et al.*^16^**	2014	Data from studies in: Argentina, Australia, Netherlands, United Kingdom, United States	Stable or stabilised unstable angina Excl. studies on acute conditions, and in-stent restenosis patients	OMT, PTCA, BMS, DES, CABG	Death, MI	Meta-analysis	1 year, 3 years
**Fidan *et al.*^16^**	2007	England and Wales	AMI, secondary prevention after AMI, stable angina, unstable angina	36 condition- treatment scenarios	LY	Cohort-based model (IMPACT).	10 years
**Griffin *et al.*^18^**	2007	England	ACRE cohort. No exclusion criteria. Both stable and acute presentations Patients rated as clinically appropriate for CABG, PCI or both	PCI, CABG, OMT	QALY	Trial-based (prospective observational) regression analyses	6 years
**Hlatky *et al.*^19^**	2009	United States, Canada, Brazil, Mexico, the Czech Republic, and Austria	Stable coronary artery disease. Diabetes	OMT, PCI, CABG	LY, QALY (sensitivity analysis only)	Trial-based (RCT) regression & survival analyses	4 years, lifetime
**Vieira *et al.*^20^**	2012	Brazil	Stable multi-vessel disease with normal systolic ventricular function	OMT, PCI, CABG	QALY, event- free survival	Trial-based (RCT) survival analyses	5 years

* ACRE = Appropriateness Coronary REvascularisation, BMS = bare metal stent, CABG = coronary artery bypass grafting, DES = drug-eluting stent, LY = life years, MI = myocardial infarction, OMT = optimal medical therapy, PCI = percutaneous coronary intervention, PTCA = percutaneous transluminal coronary angioplasty, QALY = quality-adjusted life-year, RCT = randomised controlled trial.

All studies adequately reported on the CHEERS Statement items relating: to model/analysis description, background and reasons for undertaking economic evaluations, relevant patient groups and sub-groups, comparators, time horizons and choice of health outcomes included in their analyses. The most poorly reported element related to reasons for choice of model (part of item 15). Only three studies did this. The others described the analysis undertaken, but did not give reasons for the chosen strategy.

### Usefulness of evaluations

While for the most part the studies reported their analyses and findings to a good standard when assessed against the CHEERS Statement, their usefulness to decision-makers is arguably of greater importance. The summary data extracted in relation to usefulness of each paper is shown in Table 4. Our assessment of the usefulness of the reporting for decision-makers is in Table 5.

**Table 5.** Usefulness of elements of each study for decision-makers, rated as yes, partly or no.

Author	Quality of life measure	Effect on costs	Effect on health benefits	Description of uncertainty	Policy suggestion/ direction	Overall usefulness rating
**Caruba *et al.*^16^**	No	Partly	No	No	No	Low
**Fidan *et al.*^17^**	No	Partly	Partly	No	Yes	Medium
**Griffin *et al.*^18^**	Yes	Yes	Yes	Yes	Yes	High
**Hlatky *et al.*^19^**	Partly	Yes	Partly	Partly	Partly	Medium
**Vieira *et al.*^20^**	No	Yes	No	No	Partly	Low

Of the five papers reviewed in full, three were trial-based analyses, one used a cohort modelling approach and there was one meta-analysis ( Table 4). The timeframes analysed ranged from 1 year post-intervention to a lifetime horizon. The studies came from a wide range of countries. Three of the five studies used quality of life measures in their analyses, one of which only considered QALYs as part of sensitivity analyses. The others used length of life measures to assess cost-effectiveness and one also used the clinical endpoint of myocardial infarction. All studies based their analyses on direct costs only.

In assessing the usefulness of reporting, we found that while most studies reported on various items, reporting was not always easy to interpret in the context of decision-making. In judging the reporting we were looking for a clear direction or suggestion about how the results of the analysis could be used to improve the efficiency of healthcare resource use. Only two studies made a clear statement about changing the allocation of resources or how the outcomes are relevant to policy ^17, 18^. We rated two studies as low usefulness, two medium and only one highly useful for decision-makers.

Caruba *et al.* ^16^ carried out a meta-analysis of cost-effectiveness studies. After concluding that there was no statistically significant difference between treatment strategies on clinical endpoints of myocardial infarction or death, the analysis was conducted on costs only, over 1 and 3 years ^16^. As a result, the analysis focusses primarily on cost differences across the treatments. They estimated that substantial cost savings could be made through the management of patients with stable angina, using medical therapy ^16^.

A more detailed examination of the outcomes reported by Caruba *et al.* ^16^ revealed that while no statistically significant differences were found, there appears to be some clinically significant difference in treatment effectiveness. The confidence intervals of hazard ratios reported for both death and myocardial infarction are very wide. For example, at three years follow-up, confidence intervals related to estimates of risk of death range from a halving to a doubling of risk for all comparator treatments ^16^. Similarly, the probabilities of being the best treatment vary widely; from 0.49 for drug-eluting stents to 0.05 for percutaneous transluminal coronary angioplasty (for risk of death at three years follow-up) ^16^. These results suggest both that there is a high degree of uncertainty regarding estimates of effectiveness, and therefore that a clinically significant difference between the treatments is possible. This could greatly affect estimates of cost-effectiveness. In addition to uncertainty regarding estimates of effectiveness, the authors highlight important limitations and high levels of uncertainty in the overall findings.

We rated this meta-analysis as of low use to decision-makers due to the level of uncertainty described, making it difficult to interpret how the findings might be used to direct policy or practice. While a high level of uncertainty in results should not disqualify an analysis from being useful, the authors did not make any statements which assist in determining how the findings might be used. In addition, the difference in effectiveness of treatments was not fully explored, adding even more uncertainty to the findings.

An analysis by Fidan *et al.* ^17^ modelled the life years gained for 36 condition–treatment scenarios for coronary artery disease. These included everything from acute myocardial infarction to primary prevention using statins ^17^. They used the IMPACT model; a large cell-based mortality model of coronary heart disease risk and treatment ^17, 21^. Cost-effectiveness ratios were reported, however these are not presented as incrementally, which makes it difficult to do head-to-head treatment comparisons. All treatments were examined against the baseline mortality rates, and they found that medical and surgical treatments prevented or postponed over 25,000 deaths in patients with coronary artery disease ^17^. The approach ranked different interventions, showing a 100-fold difference in cost-effectiveness across all treatments, but it does not provide insight into incremental costs associated with new technology or interventions ^10^. Again, the analysis only considers length of life, not quality of life. While this provides useful information about length of life it does not consider the full effect of different treatments. Included in this assessment are treatments for chronic angina. It is a somewhat counterintuitive approach to only examine length of life gains from a treatment that targets symptom relief.

While the reporting in this study is clear, we rated it as of medium usefulness for decision-makers because it does not include an assessment of the full effect of treatments on health outcomes (i.e. it only assessed length of life). The authors did, however, present a general policy suggestion, stating that investment in secondary prevention was likely to produce gains in length of life for lower costs.

Prior to the analysis of the MASS-II trial, there had been no cost-effectiveness analysis based on a trial comparing percutaneous intervention, surgery and medical therapy together ^20^. To address this gap, Griffin *et al.* ^18^ conducted an economic analysis using the Appropriateness of Coronary REvascularisation (ACRE) study cohort. The ACRE study rated patients as appropriate for percutaneous coronary intervention and/or coronary artery bypass grafting but followed them according to the treatment they actually received ^18^. Economic analysis of the ACRE study data concluded that coronary artery bypass grafting was cost-effective compared with percutaneous coronary intervention in patients classified as appropriate for bypass grafting only or for both bypass grafting and percutaneous intervention ^18^. The analysis also found that percutaneous coronary intervention was not cost-effective when compared with medical therapy for patients classified as appropriate for percutaneous coronary intervention only ^18^. The results of this analysis are useful because they include quality of life outcomes. However, the approach used averaged quality of life over the 6-year period using a regression model ^18^. This gives some good information about the average quality of life of patients receiving different treatments over the time period, but does not account for events during which patients might experience reduced quality of life, such as a period of hospitalisation for a subsequent procedure.

We rated this analysis as of high usefulness to decision-makers. While the estimates of quality of life could be improved, the authors make clear statements about the changes in costs and health outcomes achieved through different treatments. They also make a clear statement of how to make changes to resource allocation based on their results, which could benefit the health service. One limitation to the usefulness of the outcomes presented is that they only cover the six-year trial period. This may not be long enough to see the full cost-effectiveness of treatment for a chronic disease and the authors foresee extending the model over a lifetime horizon in future work ^18^.

The analysis by *Hlatky et al.* in 2009 examined the cost-effectiveness of revascularisation procedures in patients with type-2 diabetes, using data from the Bypass Angioplasty Revascularization Investigation 2 Diabetes trial (BARI 2D) ^19^. The BARI 2D study randomised patients with type 2 diabetes to medical therapy alone or medical therapy with immediate revascularisation (either PCI or CABG) ^22^. While the effectiveness of treatment for coronary artery disease has been shown to be affected by the presence of diabetes ^23– 26^, due to the high prevalence of type 2 diabetes in this patient population and more generally, this analysis was not excluded on grounds of being relevant only to a specific group. The rates of diabetes in other included studies are 36% in MASS II ^20^, 15% in the ACRE study ^18^, and 9 to 33% in the studies included in the meta-analysis by Caruba *et al.* ^16^. The economic evaluation of the BARI 2D study outcomes concluded that medical therapy was cost-effective compared with revascularisation (PCI or CABG), in the short-term (4 years) ^19^. When using lifetime projections of cost-effectiveness, however, medical therapy was cost-effective compared with PCI, and CABG was cost-effective compared with medical therapy ^15^.

The BARI 2D trial used a pragmatic approach which reflects the realities of clinical practice; patients undergoing revascularisation were not randomised to a particular revascularisation strategy (i.e. PCI or CABG); this was directed by clinicians ahead of randomisation to either prompt revascularisation or medical therapy ^22^. The effect of this is that patients were stratified into groups based on clinical markers of disease severity. The results of the study are therefore useful for choosing between medical therapy and PCI in patients with less severe disease, or between medical therapy and CABG in patients with severe disease. They are also only relevant to diabetic patients, however, as prevalence of type 2 diabetes is increasing globally, this is relevant to an increasing number of patients.

The overall results in the BARI 2D trial are based on length of life measures; quality of life measures were only used in sensitivity analyses. It was concluded that the quality of life measures did not affect the estimates of cost-effectiveness, based on life-years only. It is unclear why this choice was made, when quality of life measures provide a more comprehensive assessment of treatment effect. We rated this study as of medium use for decision-makers as it presents an analysis of real-world practice, but does not account for the full effect of treatment on patients’ health, by all but ignoring quality of life measures. Decision-makers wishing to know the full effect of different treatments on patient health outcomes need information beyond length of life.

Vieira *et al.* also conducted a trial-based analysis using data from the MASS II Trial (Medical Angioplasty or Surgery Study) ^20^. This was the only trial revealed in searches which randomised patients to each of the three treatment options. Its major conclusions were that medical therapy was cost-effective compared to CABG, and CABG was cost-effective compared to PCI ^20^. While this analysis did use QALYs they were not calculated using conventional health related quality of life surveys, but estimated based on the average time to event and angina free proportion of the population in each group ^20^. This is unlikely to provide good estimates of quality of life in these patients as the measurement assumes that in the period between events the patient has full quality of life and that those with angina have no quality of life. These estimates produced average QALYs of 2.07 to 2.81, over 5 years which if averaged over that time give utility weights of 0.41 to 0.56 (e.g. 2.07/5). These values are far below the estimates of 0.69 to 0.86 used in other analyses of patients with coronary artery disease ^27– 31^. Values of less than 0.5 are generally seen only in very debilitating conditions. The quality of life estimates in the MASS II study analysis therefore substantially undervalue the quality of life of patients with coronary artery disease. While the outcomes of that analysis do examine both costs and effectiveness of the three different treatments for coronary artery disease, the outcomes reported are not useful for those making decisions about resource allocation because they do not allow comparison with other areas of healthcare or report the incremental cost per QALY gained.

We rated the analysis by Vieira *et al.* ^20^ as of low usefulness to decision-makers because although quality of life was included in the analysis, it was not done in a way that makes it comparable to other studies. In addition to these novel methods of QALY estimation, the authors did not conduct incremental analyses, nor did they discuss any uncertainty in their findings.

It is worth noting that further research has been undertaken using the MASS II trial data and a validated quality of life instrument ^32^. Unfortunately, only a conference abstract was available and it was therefore not included in the analysis. The results available in that abstract show much higher average health utility weights of 0.77 to 0.81 ^32^, aligning them with the values seen in other analyses of coronary artery disease ^27– 31^. When published, the full analysis will add greatly to the current knowledge.

Endnote library of retrieved references Data related to this review are available in an EndNote Library, containing all references retrieved using the search terms described. This library also contains subfolders used to categorise papers during the review process. Click here for additional data file. Copyright: © 2018 McCreanor V et al. 2018 Data associated with the article are available under the terms of the Creative Commons Zero "No rights reserved" data waiver (CC0 1.0 Public domain dedication).

## Discussion

### Challenge for health service decision-makers

When operating under conditions of scarce resources there is responsibility to promote cost-effective care, achieving larger health gains from available resources. In an ideal world, decision-makers would have information about the long-term costs and health outcomes achievable through different configurations of health services and be able to invest accordingly. However, without good evidence of cost-effectiveness, it is impossible for decision-makers to fulfil this responsibility with any confidence.

### Current evidence and value of evidence for coronary artery disease

In the case of stable coronary artery disease, we have some information about the comparative cost-effectiveness of optimal medical therapy, PCI and CABG, but it is difficult to interpret in the context of healthcare resource allocation. Overall, the results of cost-effectiveness analyses suggest that in most scenarios optimal medical therapy is cost-effective compared with alternatives and CABG is cost-effective for certain patient groups.

However, only three of the five studies included in this review used quality of life as an effectiveness measure. This is a key outcome measure for cost-effectiveness and good decision making. For chronic diseases, improvements in quality of life are equally as relevant as improvements in length of life. In the case of coronary artery disease, relief from chest-pain is a key objective of treatment. If quality of life is not measured, two treatments affording a patient equal length of life are valued equally even where one restored the patient to better health than the other. However, if given the choice, patients and health service providers would choose the option most likely to provide the best improvements to quality of life. Therefore, analyses based solely on length of life measures do not provide a full picture of the effectiveness of each treatment.

Another omission from the literature is to neglect the lifetime costs and health outcomes. Important information about the longevity of treatment effect may be overlooked. This is particularly important for chronic diseases such as coronary artery disease, where important costs and health consequences are missed when they occur beyond the timeframe of a clinical trial.

### Confidence in changing services based on current evidence

The current information about cost-effectiveness of treatments for stable coronary artery disease suggests that either optimal medical therapy or CABG could be cost-effective, over a 1-year to lifetime timeframe. There is no evidence from the papers included in this review that PCI is cost-effective when compared with other competing treatment options. Therefore the current cost-effectiveness evidence does not support the increased use of PCI that has been seen in Australia and internationally, and there is increasingly reduced evidence of clinical effectiveness ^33^.

However, it is unlikely that healthcare decision-makers would be confident making changes to the allocation of resources based on the economic evidence outlined in this review. Our evaluation of the relevant cost-effectiveness evidence showed that overall, information is not presented in a way useful for decision-making. We found only one study to be of high usefulness in this context ( Table 5).

Our assessment of the usefulness of the cost-effectiveness studies examined suggests that poor reporting may contribute to the problem. We rated two out of the five studies as ‘low usefulness’ for decision-makers. Reporting was either too complex, making interpretation challenging, or uncertainty was not reported in way that made clear the effect of acting on the evidence. It is also apparent from our analysis, that the CHEERS Statement, while encouraging comprehensive reporting, is not sufficient alone, to assess the usefulness of economic evaluations.

Others have explored barriers to the use of economic evaluation by decision-makers ^34^. A review by Merlo *et al.* ^34^ used an accessibility and acceptability framework developed by Williams and Bryan ^35^ to categorise barriers to use of economic evaluations. Accessibility refers to the ability of decision-makers to interpret and use economic evidence, and includes issues of complexity and timeliness of economic evaluations ^34, 35^. Acceptability includes factors associated with scientific rigor, applicability to the institution in which decisions are to be made, and ethical considerations such as equity ^34, 35^.

While some studies we examined included comprehensive reporting, they did not always present results in a way conducive to decision-making. For example reporting large results tables covering many different clinical scenarios, as seen in the paper by Fidan *et al.* ^17^, demonstrates the complexity but does nothing to assist those wanting to make higher-level resource allocation decisions. While clinical complexity is inherently important in many contexts, for it to be of use for decision-making, it needs to be summarised in a way which makes clear the likely impact of acting on the evidence presented. In fact, presenting all clinical complexities can mean the overall message is lost in the details, making research less accessible to decision-makers and decreasing the chance that any improvement to health services will follow. This is unfortunate, considering the purpose of economic evaluation is to provide evidence for resource allocation decisions to improve service delivery.

Most of the economic evaluations we have assessed suffer from a number of accessibility problems which prevent them from being useful for decision-making; the interpretation of results and their applicability to policy are generally lacking. The information in Table 5 reveals that only one study, by Griffin *et al.* ^18^, included a clear expression of the confidence in estimates of cost-effectiveness, useful in the context of decision-making. Hlatky *et al.* ^19^ made a less-clear statement. Only two studies, Griffin *et al.* ^18^ and Fidan *et al.* ^17^, made clear statements of what direction policy should take based on results of their research (Htlaty *et al.* ^19^ and Vieira *et al.* ^20^ expressed a direction but less-clearly). The result of this highly complex reporting and lack of clear policy direction to follow, is that some otherwise very scientifically rigorous analyses have failed to generate any policy changes or perhaps even reach their intended audience.

In addition to these complexities, while the patient populations may be similar to those in Australia, none of these studies have been carried out in the Australian context. For the purposes of resource allocation decisions in Australia, an analysis based at least on Australian costs, is required.

### Conclusion

The evaluation of the studies in this review highlights a lack of information useful for making decisions about the allocation of resources for coronary artery disease. It is concerning that over $2 billion of Australia’s annual healthcare budget is being spent on coronary artery disease, with inadequate economic evidence. Since the mid to late 1990s, increased spending has been directed towards PCI with stenting, over coronary artery bypass grafting, but there is insufficient economic evidence to support this transition. Compounding this, the current cost-effectiveness evidence which would suggest a move away from PCI and stents remains too unclear and uncertain for policy-makers to be confident in making changes. In a time of increased pressure on health budgets, economic evidence should be fundamental to resource allocation decisions.

For those wishing to make resource allocation decisions to improve the efficiency in treatment of coronary artery disease, the current evidence is insufficient. A transparent, structured, lifetime analysis of all competing treatments, incorporating quality of life measures, would be valuable for decision makers. The analysis should account for fluctuations in the quality of life of patients over their lifetimes, related to symptom relief, repeat procedures and acute events.

The findings of recent trials, in particular ORBITA ^33, 36^ and ISCHEMIA ^37^, will strengthen the evidence about the effectiveness of conservative versus invasive therapy for stable coronary artery disease. In turn these data will enable improved cost-effectiveness analyses.

To be of use to decision-makers and have a better chance of generating policy change, analyses must be accessible; economic evaluations should include a clear indication for the direction of policy or changes to practice that should follow and a statement of the probability that such changes will the generate the predicted improvements. In their systematic review of barriers and facilitators to use of evidence by policymakers, Oliver *et al.* named clarity, relevance and reliability as some of the top barriers to use of evidence ^38^. For decision-makers to be able to act on the economic evidence, the expected effect of making changes based on the results needs to be clear. In cases where there is too much uncertainty, a strategy to improve the analysis should be outlined. We suggest that to improve reporting of economic evaluations for decision-making, an additional item could be included in the CHEERS Statement, relating to implications for policy and practice. Ideally, this would be a statement describing the implications of acting on the evidence presented; encompassing both expected improvements to health outcomes and confidence in the effect.

## Data availability

The data referenced by this article are under copyright with the following copyright statement: Copyright: © 2018 McCreanor V et al.

Data associated with the article are available under the terms of the Creative Commons Zero "No rights reserved" data waiver (CC0 1.0 Public domain dedication). 

**Dataset 1: Endnote library of retrieved references** - Data related to this review are available in an EndNote Library, containing all references retrieved using the search terms described. This library also contains subfolders used to categorise papers during the review process. 10.5256/f1000research.13616.d190562 ^39^

## Supplementary material

**Supplementary file 1:** Search terms used in each database searched.

**Supplementary file 2:** Completed PRISMA Checklist.

**Supplementary file 3:** CHEERS Statement Checklist – completed for reviewed studies.
