# Supplementary material for: A systematic review and critical analysis of cost-effectiveness studies for coronary artery disease treatment
Source: F1000Res. 2018 Jul 3;7:77. Originally published 2018 Jan 17. [Version 2] doi: 10.12688/f1000research.13616.2 (PMC6039943; doi:10.12688/f1000research.13616.2)
Supplement: Supplementary file 2 [file f1000research-7-16858-s0001.tgz › 385e2c3e-5f14-4790-8774-58ed3f543673.docx]

# Search terms for databases searched

Search terms used for CINAHL

( (MH "Costs and Cost Analysis+") ) AND ( (MH "Stents+") OR (MH "Angioplasty, Transluminal, Percutaneous Coronary") ) AND (MH "Coronary Artery Bypass+") AND ( (medical OR conservative) N5 (therapy OR treatment) OR 'secondary prevention' OR 'primary prevention') )

Search terms used for Scopus

( INDEXTERMS ( [cost effectiveness analysis] OR [cost utility analysis] ) AND INDEXTERMS ( [percutaneous coronary intervention] OR stent ) AND INDEXTERMS ( [coronary artery bypass graft] ) ) AND ( ( ALL ( ( medical OR conservative ) W/5 ( therapy OR treatment ) ) OR ALL ( ( primary OR secondary ) W/2 prevention ) ) )

Search terms used for EconLit

(("Percutaneous coronary intervention" OR PCI OR Stent) AND ("Coronary artery bypass" OR CABG OR "Coronary artery by-pass")) AND (((medical OR conservative) NEAR/5 (therapy OR treatment)) OR ((primary OR secondary) NEAR/2 prevention)) AND ("economic evaluation" OR "economic model" OR "economic modelling" OR "economic modeling" OR "economic analysis" OR "cost effective" OR "cost-effective" OR "cost effectiveness" OR "cost-effectiveness" OR "cost benefit" OR "cost-benefit" OR "cost utility" OR "cost-utility")

Search terms used for Embase

'percutaneous coronary intervention'/exp OR 'stent'/exp AND 'coronary artery bypass graft'/exp AND ('cost effectiveness analysis'/exp OR 'cost utility analysis'/exp) AND ((medical OR conservative) NEAR/5 (therapy OR treatment) OR 'secondary prevention' OR 'primary prevention')
