# Supplementary material for: A systematic review and critical analysis of cost-effectiveness studies for coronary artery disease treatment
Source: F1000Res. 2018 Jul 3;7:77. Originally published 2018 Jan 17. [Version 2] doi: 10.12688/f1000research.13616.2 (PMC6039943; doi:10.12688/f1000research.13616.2)
Supplement: Peer review history

# Peer review: Referee response for version 2

**DOI:** 10.5256/f1000research.16858.r35690

**Published:** 2018-07-10

**Authors:** James P Howard

**Affiliations:** 1 International Centre for Circulatory Health, National Heart and Lung Institute, Imperial College London and Imperial College Healthcare NHS Trust, London, UK

I'm confident that the author has dealt with all of the issues identified during peer review. I have no further comments to make.

I have read this submission. I believe that I have an appropriate level of expertise to confirm that it is of an acceptable scientific standard.

# Peer review: Referee response for version 1

**DOI:** 10.5256/f1000research.14791.r34101

**Published:** 2018-05-31

**Authors:** James P Howard

**Affiliations:** 1 International Centre for Circulatory Health, National Heart and Lung Institute, Imperial College London and Imperial College Healthcare NHS Trust, London, UK

This is an excellent review of cost-effectiveness studies comparing percutaneous coronary intervention (PCI), coronary artery bypass grafting (CABG) and optimal medical  (OMT) therapy for stable coronary artery disease (CAD). It is well-written, easy to follow, and the interpretations are sound.

 My only query about the design of the study is I'm not entirely sure why studies comparing only PCI vs OMT were excluded, given the authors go on to rightly raise concerns about a lack of high-quality data. We must remember that the majority of of patients with stable CAD are not candidates under current guidelines for CABG (e.g. if they lack 3 vessel or proximal LAD disease) and so to argue a cost-effectiveness study should use data where all three are compared because CABG could be a superior options to PCI seems strange to me. Even if CABG was deemed more effective than PCI in a study, the finding would not be applicable to most of our patients, as only a small subset of our patients would be eligible for randomisation in whatever study that data was based on.

 I also wonder whether the authors think this data will change in the next few years? The two most high profile studies of stable CAD in the last few years are probably FAME-2 and ORBITA. The former has shown improvements when measured as a reduction in a composite of death, myocardial infarction and urgent revascularisation (although this latter largely powers this and there have been significant concerns about this endpoint in unblinded settings). ORBITA initially showed no benefits in improvements in exercise time from PCI versus optimal medical therapy, but the recent secondary analysis in Circulation showed a significant reduction in the number of patients reporting angina following PCI versus placebo (when all patients were on OMT). I appreciate that the authors are reviewing cost-effectiveness studies, and it may be several years before these new data are included in such studies, but it appears that under this current design (including only cost-effectiveness studies involve CABG) would mean the only **blinded** trial of PCI versus OMT that I am aware of would be ineligible for analysis?

 Some minor points are there isn’t discussion about the role of direct versus indirect cost measurements in the different studies. This is crucial when considering the applicability of results to different healthcare settings, where sometimes indirect costs are borne by providers but sometimes not.

 Also, the authors do query about the applicability of data to Australia. Whilst this may be an issue, it seems likely that evidence from Europe and USA are of relatively-good applicability to Australia as similarly developed nations with, in some cases, similar healthcare systems.

 Finally, a tiny point is I think it would be useful to order tables 4 and 5 the same way; I foolishly initially assumed that the top study in both (the most recent study using meta-analysis in table 4 and the high quality study in table 5) were the same study, when actually the most recent study was deemed of 'low' usefulness). Indeed, table 5 may be better as a traffic light system with colour coding to make things more clear, but I will leave that up to the authors and editorial team.

I have read this submission. I believe that I have an appropriate level of expertise to confirm that it is of an acceptable scientific standard, however I have significant reservations, as outlined above.

# Response

**Published:** 2018-06-26

**Authors:** Victoria McCreanor

**Affiliations:** Queensland University of Technology, Australia

We agree with the comments regarding appropriateness of treatment in different patient groups. However, the problem with reviewing papers comparing two treatments is complexity. This would introduce three additional reviews (PCI v CABG, OMT v CABG and OMT v PCI) - All are relevant but of course, by necessity, only pertaining to more and more selected groups. Thus, comparisons would be hard to interpret, if not, impossible.

Regarding the comments about data changing in the coming years, in short, yes, we think data are likely to change.  Invasive procedures for stable disease are currently being subject to increasing scrutiny, particularly in the wake of ORBITA, as you suggest. The secondary analysis of ORBITA provides a well-needed and important contribution to the field and it will be interesting to see how those results affect cost-effectiveness estimates. We have included a comment in the discussions to that effect.  

Other important data will come from the ISCHEMIA trial, which randomises patients with stable disease to conservative or invasive strategies, particularly in relation to long-term outcomes.

In response to the other points, all studies used direct costs and we have updated the text accordingly.

We agree that evidence from Europe and USA is of relatively-good applicability to Australia and have updated the text to include a comment that existing analyses could be applied to the Australian context if Australian costs were used.

We thank you for the useful suggestion to make the tables clearer. We have updated tables 4 and 5 so the studies are in the same order and included a traffic light system for overall usefulness.

# Peer review: Referee response for version 1

**DOI:** 10.5256/f1000research.14791.r30777

**Published:** 2018-03-02

**Authors:** Elizabeth A. Geelhoed

**Affiliations:** 1 School of Allied Health, University of Western Australia, Perth, WA, Australia

This paper presents a valuable critical analysis of cost-effectiveness studies and highlights serious issues in relation to potential inefficiencies in funding for Coronary Artery Disease (CAD) treatment. As identified by the authors, heart disease is a significant area of expenditure for health in Australia and this paper contributes important information for policy consideration.

 The paper is well-written and raises a number of recommendations useful for general presentation of cost-effectiveness analyses in the literature. In particular, these recommendations are useful in terms of the quality of life measurement and the application of the CHEERS checklist.

 One interesting issue raised here – which could potentially be further explored - is in terms of high-level policy changes. All new procedures presented for listing on Medicare or the PBS necessarily undergo a rigorous process of evaluation, including assessment of cost-effectiveness evidence, which often includes commissioned modelling studies to apply international clinical findings for Australian scenarios. The economic evaluation requires comparison of the new technology or drug with the status quo (or best current alternative) to appraise the incremental cost-effectiveness for the specific population group defined in the submission. However the point raised here is an important one – that incremental benefits and costs in cost-effectiveness may not reflect the bigger picture if the underlying comparator is flawed. Nonetheless it is a much greater undertaking to address wider policy ramifications, particularly where evidence continues to accrue. (Notably the current MBS taskforce is endeavouring to address such challenges.)

 One major criticism however is that it is not made clear to the reader that the analysis specifically targets stable angina, which is a subgroup of CAD patients. In particular, the abstract suggests that the results apply to treatment of all CAD patients and hence the results are potentially misleading when considered across all patients with CAD. It is important to know what proportion of CAD patients present with stable disease (and hence are eligible for all three treatments (CABG, PCI, MT). The information is implicit since it is clear from the population group of each of the included trials that patients with acute disease have been excluded. The fairly recent RCT which provides the most enlightening data, has quite strict exclusion criteria, which lists a number of presentations. The conclusions of the review are valid, but the subgroup requires transparency.

 This leads to a second point, which questions the importance of the clinical diagnosis. NICE clinical guidelines recommend drug treatment for stable angina unless symptoms are not satisfactorily controlled, in which case interventional procedures should be considered. This suggests a value judgement in selecting patients for interventional care. Does this potentially present a continuum that has shifted over time? It is important to assess what proportion of the cited 57% increase in PCI has been for the treatment of stable CAD. A related query is whether the non-RCT studies were subject to possible selection bias.

 A fundamental question then arises as to whether the cost-effectiveness studies should stand apart from the clinical data. That is, if the clinical evidence is changing (and clearly the controversy around interventional procedures for stable disease reflects the growing evidence) – it may be most useful for the cost-effectiveness to be presented for the specific sub groups that are most controversial – for example based on the differential burden of ischaemia.  

 Queries and comments:

 ·       Reviews were excluded from consideration – were they considered for comparison of findings and were they scrutinised for potentially missed references?

 ·       ‘..most of the .. papers did not examine medical therapy.’ Was this because they pertained to acute presentations or was medical therapy overlooked for stable presentation?

 I disagree with some of the statements made around usefulness for policy. For example the statement that presenting clinical complexities ‘does nothing to assist those wanting to make higher level resource allocation decisions’. Complexity may limit relevance in some cases, but if cost-effectiveness varies according to clinical diagnosis (and evidence supports this), then the heterogeneity may be an important consideration. Also, the statement referring to publications that consider only 2 treatments as not being useful to policy – if the policy decision relates to critical cases such as myocardial infarction or if it relates to a minor change to policy already in existence, then comparison of more than 2 options may not be relevant. Thirdly, non-Australian evidence may be appropriate if the clinical findings are relevant to an Australian population and Australian cost data can be applied.

 It may be in the wording of some of these statements, given the separation of higher level allocation decisions and those relating to small changes in resource allocation. However most policy decisions assess a well-defined intervention, compared to a current alternative, for a clearly defined patient group (which is necessary to prevent leakage), so commonly the detailed breakdown of cost-effectiveness provides critical information for those decisions.

 The paper provides an interesting supplement to the current clinical literature on the controversy around treatment of stable coronary artery disease. The discussion of the importance of policy-relevant evidence will inform future cost-effectiveness analyses of trials currently in progress.

I have read this submission. I believe that I have an appropriate level of expertise to confirm that it is of an acceptable scientific standard.

# Response

**Published:** 2018-06-26

**Authors:** Victoria McCreanor

**Affiliations:** Queensland University of Technology, Australia

We agree with these comments regarding the Australian regulatory system, however, we don’t feel this paper is the right place for a discussion of Australian regulatory processes.

 We also agree that economic evidence may change as new evidence accrues, however, cost-effectiveness analysis based on imperfect information should still be used as long the caveats are noted and the decision makers are fully informed about the problems with any analysis. Using imperfect information to make decisions is better than making them with no information. As with clinical evidence and practice, economic evaluations can be updated as new evidence becomes available.

 We have updated the text to make it clear that the review relates to patients with stable disease.

 We agree with the comments regarding the importance of clinical diagnosis, however there is mounting evidence, for example from the first results from the ORBITA trial published in the Lancet, that in stable patients, interventional treatment may not improve patient outcomes to the extent originally expected.

 Regarding the change in preference towards PCI, we have updated the text to include the proportion increase in PCI related to non-AMI patients.

 The point raised about non-RCT studies being subject to selection bias is valid, however we would suggest that perhaps these better reflect real-world practice and therefore may be more useful for cost-effectiveness analysis and policy than results from highly-controlled clinical trials. RCTs aim for high internal validity, which comes at the expense of generalisability, and generalisability is more important for economic evaluation and policy decisions.

 We agree that where clinical evidence changes, economic analyses may also need to be updated. However, we think it is important to use the evidence currently available, rather than waiting for perfect information.

 We agree that where the effectiveness is affected by particular clinical sub-groups, it will likely be important to present the cost-effectiveness evidence accordingly. In this paper we only attempted to review cost-effectiveness analyses which compared PCI, CABG and OMT in groups of patients where all three were considered appropriate treatment options.

 The other reviewer also raised queries about relevance across different patient sub-groups and we noted the following: This would introduce three additional reviews (PCI v CABG, OMT v CABG and OMT v PCI) - All are relevant but of course, by necessity, only pertaining to more and more selected groups. Thus, comparisons would be hard to interpret, if not, impossible.

 In response to the queries and comments:

 We have updated the text to note that we examined review papers for potentially missed references.

 The sentence referred to, ‘.. *most of the .. papers did not examine medical therapy.’*, meant that most studies did not examine all three treatment groups together. However, most compared interventional treatments with each other and did not consider medical therapy as an alternative treatment on its own. Some pertained to acute presentations and others to stable, however, as noted this paper relates only to stable disease.

 In response to comments about statements regarding usefulness for policy:

 We agree that clinical complexity is important in many instances. In the comment highlighted, we refer specifically to the Fidan et al paper, which presents 34 scenarios together, making it difficult to interpret in the context of resource allocation decision-making. We have updated the text to clarify this.

 Regarding comments about two-treatment comparisons not being useful for policy, we agree that if only two treatments are relevant to a certain cohort, then an analysis of only those two would be useful. We have also addressed in this in an earlier comment.

 Perhaps it is our wording, but we do not mean to imply that two-treatment comparisons are irrelevant in all contexts. We do state that if a two-treatment analysis omits another relevant treatment, then the analysis is limited.

 We agree with the comments regarding the Australian context and have updated the text to note that Australian costs could be applied to an existing analysis. 

 We agree that detailed breakdown of cost-effectiveness analysis and caveats is important for policy decisions and have discussed this in some of our earlier comments. Our main point, however, is that presenting too much detail can detract from the overall message.
